# Supplementary material for: Efficient molecular doping of polymeric semiconductors improved by coupled reaction
Source: Nat Commun. 2024 Jul 12;15:5854. doi: 10.1038/s41467-024-50293-1 (PMC11245478; doi:10.1038/s41467-024-50293-1)
Supplement: Supplementary file 1 — Supplementary Information [file 41467_2024_50293_MOESM1_ESM.pdf]

Efficient molecular doping of polymeric semiconductors improved by coupled reaction

Jiahao Pan[1], Jing Wang[1,2], Kuncai Li[1], Xu Dai[1], Qing. Li[3], Daotong Chong[2], Bin Chen[2], Junjie Yan[2], Hong Wang[1,2]*

[1] State Key Laboratory of Multiphase Flow in Power Engineering & Frontier Institute of Science and Technology, Xi'an Jiaotong University, Xi'an, 710054, China

[2] School of Energy and Power Engineering, Xi'an Jiaotong University, Xi'an, 710054, China

[3] College of Chemistry and Chemical Engineering, Dezhou University, Dezhou, 253023, Shandong, China

E-mail: hong.wang@xjtu.edu.cn

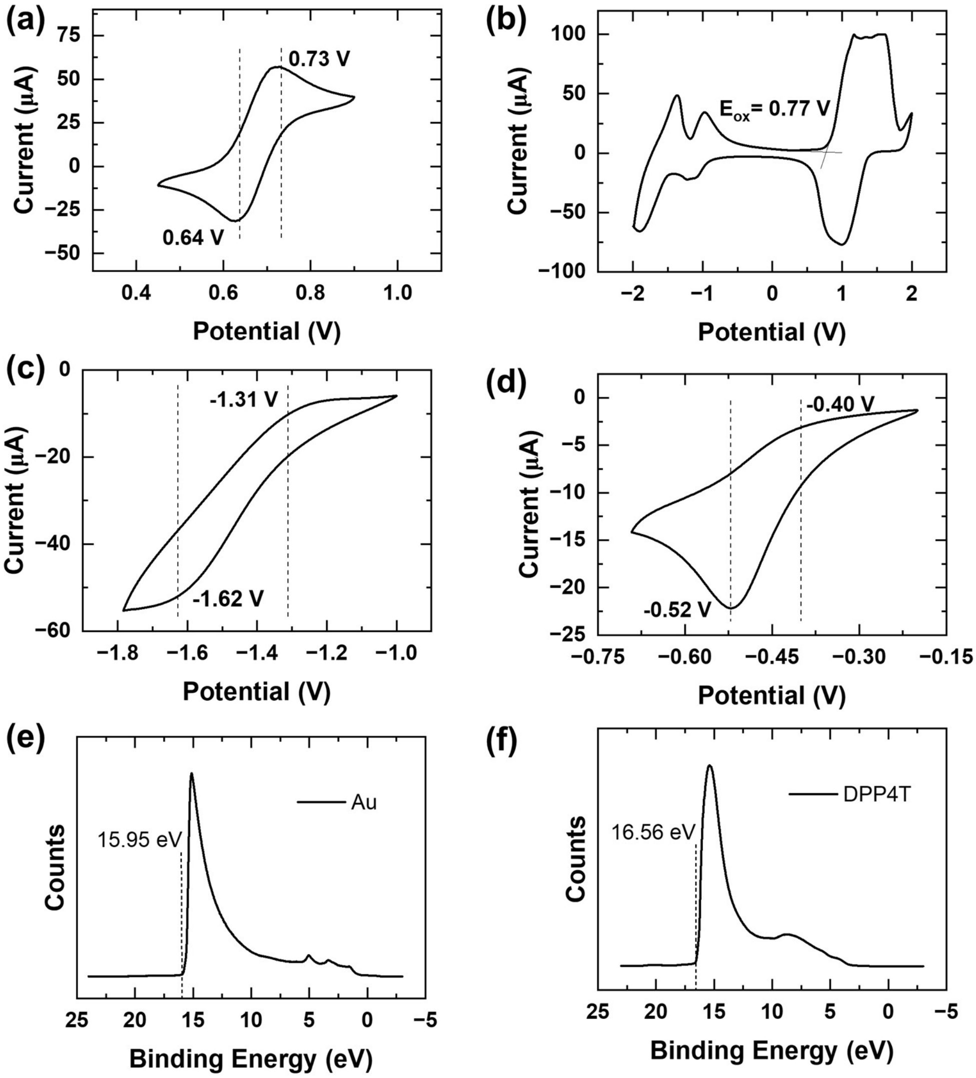

Figure S1. Cyclic voltammetry curve of (a) Cp_2Fe, (b) DPP4T, (c) TEMPO(D1), and (d) $B(C_6F_5)_3$(A1); UPS spectrum of Au(e) and DPP4T(f).

In general, EA and IP values can be used to predict whether doping reactions occur. For p-type doping, the EA value of the dopant is often higher than the IP value of the polymer to ensure an efficient molecular doping.[1,2] However, in this work, the EA value of the dopants is lower than the IP value of the polymer, which still leads to efficient p-type doping due to the coupled reaction.

Additional experiments were performed to evaluated the EA and IP of the dopants and polymers with an electrochemical method according to the following equation (1) and (2).[3]

$$E_{\mathrm{HOMO}} = -IP = -e(\varphi_{\mathrm{ox}} - \varphi_{\mathrm{Fc^+/Fc}} + 4.8)\ (\mathrm{eV}) \quad (1)$$

$$E_{\mathrm{LUMO}} = -EA = -e(\varphi_{\mathrm{red}} - \varphi_{\mathrm{Fc^+/Fc}} + 4.8)\ (\mathrm{eV}) \quad (2)$$

The obtained experimental IP and EA values were shown in Table S1, which matched well with the theoretical values acquired using Gaussian for DPP4T, A1($B(C_6F_5)_3$, D1(TEMPO). In addition, the IP value of DPP4T was further evaluated by ultraviolet photoelectron spectroscopy (UPS) which also agreed well with the theoretical IP value (Table S1). Similar IP and EA values were reported in previous literature for DPP4T (IP: 4.97-5.2 eV),[4-7] A1(EA: 3.03-3.31 eV),[8] D1(2.33 eV).[9]

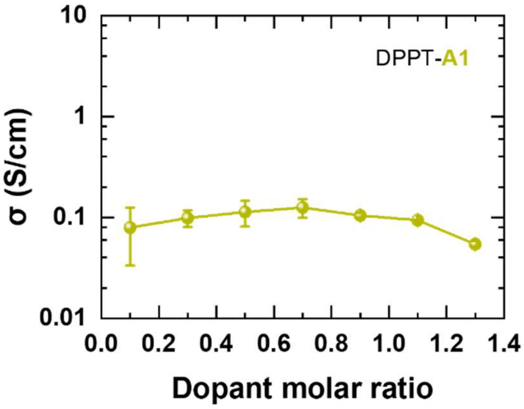

Figure S2. Electrical conductivity of BCF doped DPP4T (DPP4T-A1) as a function of dopant molar ratio. Error bars were standard deviations from at least 3 sample.

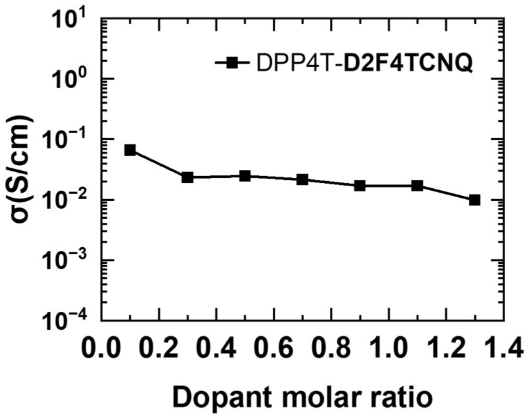

Figure S3. The electrical conductivity of DPP4T-D2F4TCNQ varies with dopant molar ratio. Error bars were standard deviations from at least 3 sample and too small to be seen in this figure.

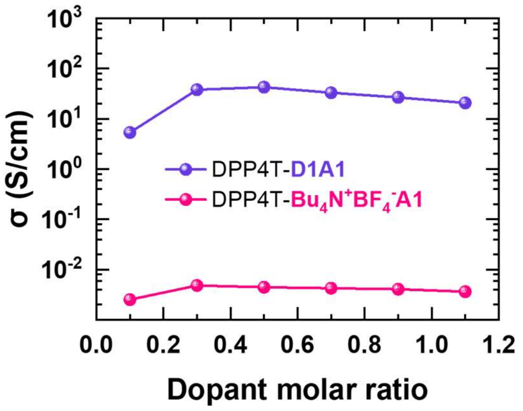

Figure S4. Electrical conductivities of BCF+TEMPO$^+$$BF_4^-$ doped DPP4T and BCF-$Bu_4N^+BF_4^-$ doped DPP4T as a function of dopant molar ratio. Error bars were standard deviations from at least 3 sample and too small to be seen in this figure.

BCF-$Bu_4N^+BF_4^-$ doped DPP4T is unable to exhibit better doping results to than dopant TEMPO$^+$$BF_4^-$ or additive BCF doped DPP4T, indicating that the existence of TEMPO$^+$ cation is the main reason for improving the doping effect of in DPP4T-D2A1 films.

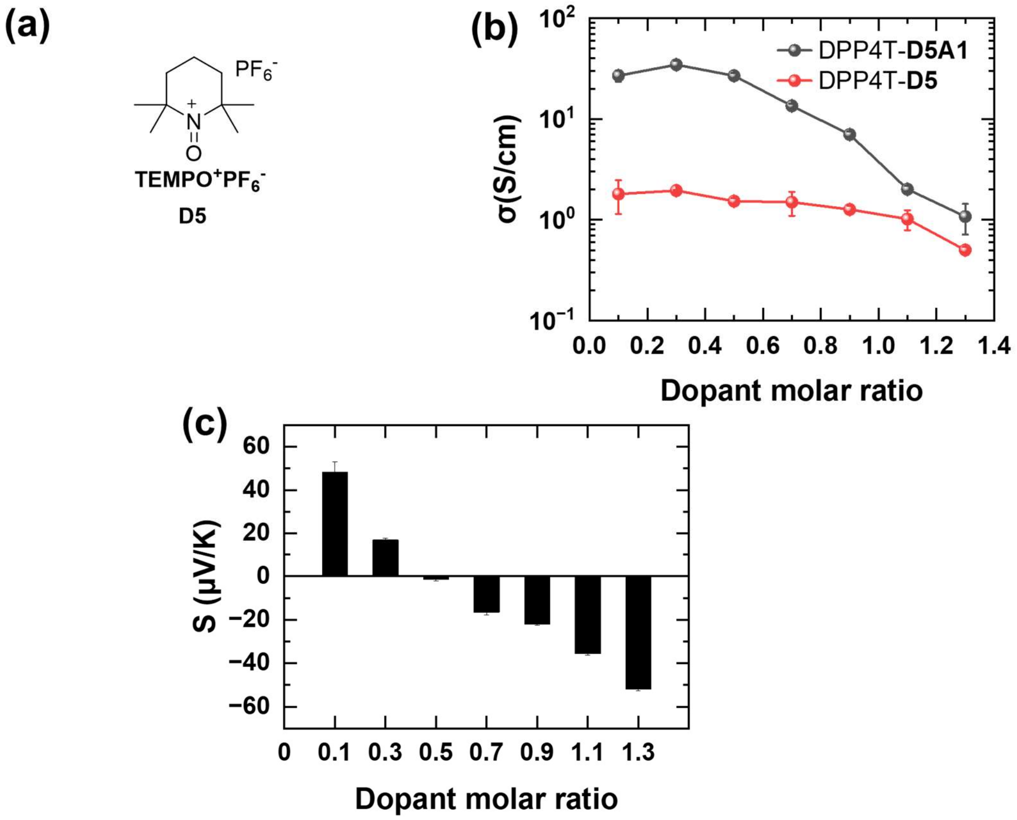

Figure S5 Coupled reaction doping of DPP4T-D5A1. (a) Chemical structure of D5; (b) The conductivity of DPP4T-D5A1 varies with dopant molar ratio; (c) The Seebeck coefficient of DPP4T-D5A1 varies with dopant molar ratio. Error bars were standard deviations from at least 3 sample.

2,2,6,6-tetramethyl-1-oxopiperidinium hexafluorophosphate ($TEMPO^+PF_6^-$, D5) was used as dopant with additive A1 and polymer DPP4T. The result showed that significant improvement could also be obtained while doping DPP4T with D5A1 (DPP4T-D5A1) with a maximum electrical conductivity of 34.5 S/cm and the polarity of Seebeck coefficient also changed from p- to n-type after increasing the dopant molar ratio up to >0.5. The results were similar to that of DPP4T-D2A1, which demonstrated that the anion BF_4^- is not specific of the coupled reaction doping process.

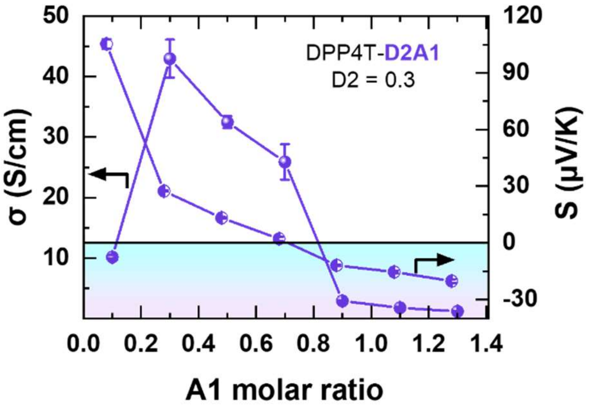

Figure S6. Electrical conductivity and Seebeck coefficient of DPP4T-D2A1 film as a function of additive A1 molar ratio when D2 molar ratio maintains at 0.3. Error bars were standard deviations from at least 3 sample.

DPP4T-D2A1 film has the maximum electrical conductivity with the D2: A1 molar ratio of 0.3: 0.3. We started doping DPP4T with D1(D2) and A1 at the dopant-to-additive ratio of 1:1 because literature indicated that the formation of $D1^-$ and A1 at the ratio of 1:1 (J. Am. Chem. Soc., 2017, 139, 10062). Then, the electrical conductivity as a function of the dopant molar ratio was tested as shown in Figure 3a of the original manuscript. Dopant molar ratios of 0.1, 0.3, 0.5, 0.7, 0.9, 1.1, 1.3 were used casually. It was found that a significant improvement in terms of the electrical conductivity was observed at the dopant molar ratio of 0.3 in both DPP4T-D1A1 and DPP4T-D2A1 films. After that, the dopant-to-additive ratio was then screened with the fixed D2 molar ratio of 0.3 for DPP4T-D2A1 films as shown in Figure S6. Because the electrical conductivity of DPP4T-D2A1 film was higher than that of DPP4T-D1A1 film. The A1 molar ratio of 0.1, 0.3, 0.5, 0.7, 0.9, 1.1, 1.3 were used casually. The results indicated that the maximum electrical conductivity was obtained when the A1 molar ratio equaled to the D2 molar ratio (dopant-to-additive ratio 1:1).

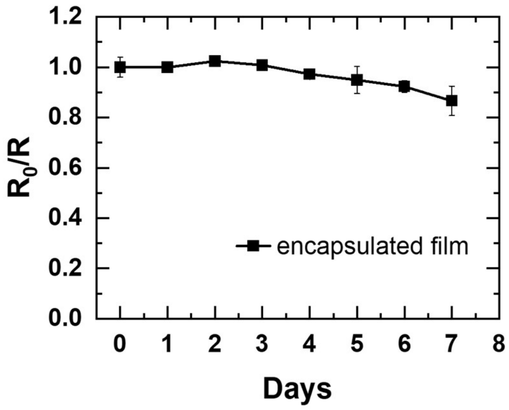

Figure S7. The electrical conductivity of laminated DPP4T-D2A1 at 0.3 dopant molar ratio as a function of time. Error bars were standard deviations from at least 3 sample.

The electrical conductivity of DPP4T-D2A1 at 0.3 dopant molar ratio dropped fast while being kept in the ambient at room temperature, which became less than half of the initial value within 24 h. However, these films exhibited a good stability while they were laminated by polyethylene terephthalate (PET) films. The electrical conductivity of DPP4T-D2A1 film at 0.3 dopant molar ratio could be maintained >85% for 7 days while being laminated by PET films.

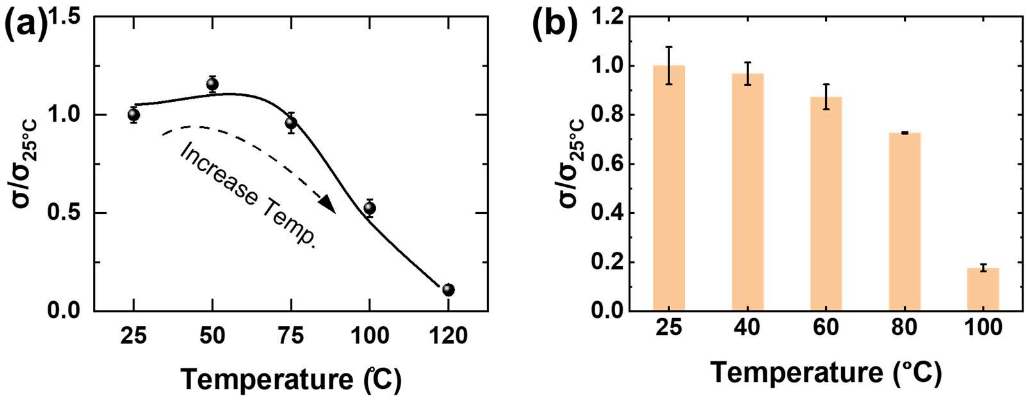

Figure S8. Temperature effect on the coupled reaction doping. (a) Temperature dependent electrical conductivity of DPP4T-D2A1 films at the temperature range of 25˚C-120˚C; (b) The electrical conductivities of DPP4T-D2A1 films prepared at different temperatures. Error bars were standard deviations from at least 3 sample.

The temperature dependent electrical conductivity of DPP4T-D2A1 films at dopant molar ratio of 0.3 in Figure S8a was obtained with the commercial equipment NETZSCH SBA-458 (Germany). The electrical conductivities were measured with a four-probe method under argon protection. The temperature rise rate was 0.5 K/min. The total measurement time was about 3 h while increasing the temperature from 25°C to 120°C.

The electrical conductivity varying with the doping temperature in Figure S8b was measured with DPP4T-D2A1 films that were obtained by drop-casting a mixture of polymer, dopant and additive (dopant molar ratio is 0.3) after stirring and reacting at different temperatures for 2 hours under N2 protection in the glove box. After the films dried at room temperature under N2 protection in the glove box, their electrical conductivities were measured with NETZSCH SBA-458 (Germany) by using four-probe method under argon protection at room temperature.

The temperature dependent electrical conductivity of DPP4T-D2A1 films was tested at the temperature range of 25˚C-120˚C. It indicated that the electrical conductivity maintained when the temperature was lower than 75˚C. After that the electrical conductivity of DPP4T-D2A1 films decreased, which was only 11% at the 120˚C (Figure S8a).

The obtained p-doping films in this work exhibited a good thermal stability, which was better than the high EA value dopant $FeCl_3$ doped polymer.[10-12] The electrical conductivity of the $FeCl_3$ doped polymer decreased to ~50% of the electrical conductivity measured at room temperature after being kept at 60°C.[10] The thermal stability was comparable to the low EA value dopant F4TCNQ (2,3,5,6-tetrafluoro-7,7,8,8-tetracyanoquinodimethane) doped polymers.[13] The results indicated that the coupled reaction doping method was promising for the preparation of efficiently doped and temperature stable conducting polymers for organic electronics by avoiding the utilization of high EA value dopants.

The film preparation temperature was also optimized. It showed that DPP4T-D2A1 films prepared at room temperature exhibited the highest electrical conductivity which should be due to the poor stability of TEMPO- at higher temperature (Figure S8b).

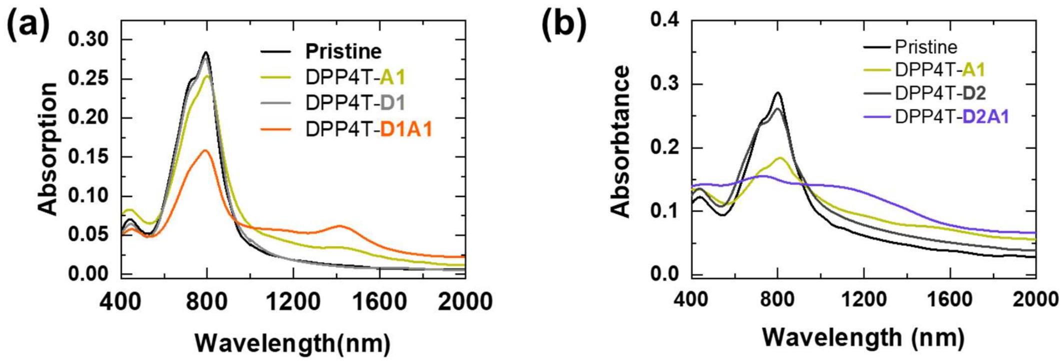

Figure S9. UV–vis–NIR spectra of pristine DPP4T film and doped DPP4T-D1, DPP4T-A1, DPP4T-D1A1 film at each maximum electrical conductivity (a); UV–vis–NIR spectra of pristine DPP4T film and heavily doped DPP4T-A1, DPP4T-D2, DPP4T-D2A1 film with dopant molar ratio of 1.1 (DPP4T-D2A1 has a negative Seebeck coefficient) (b).

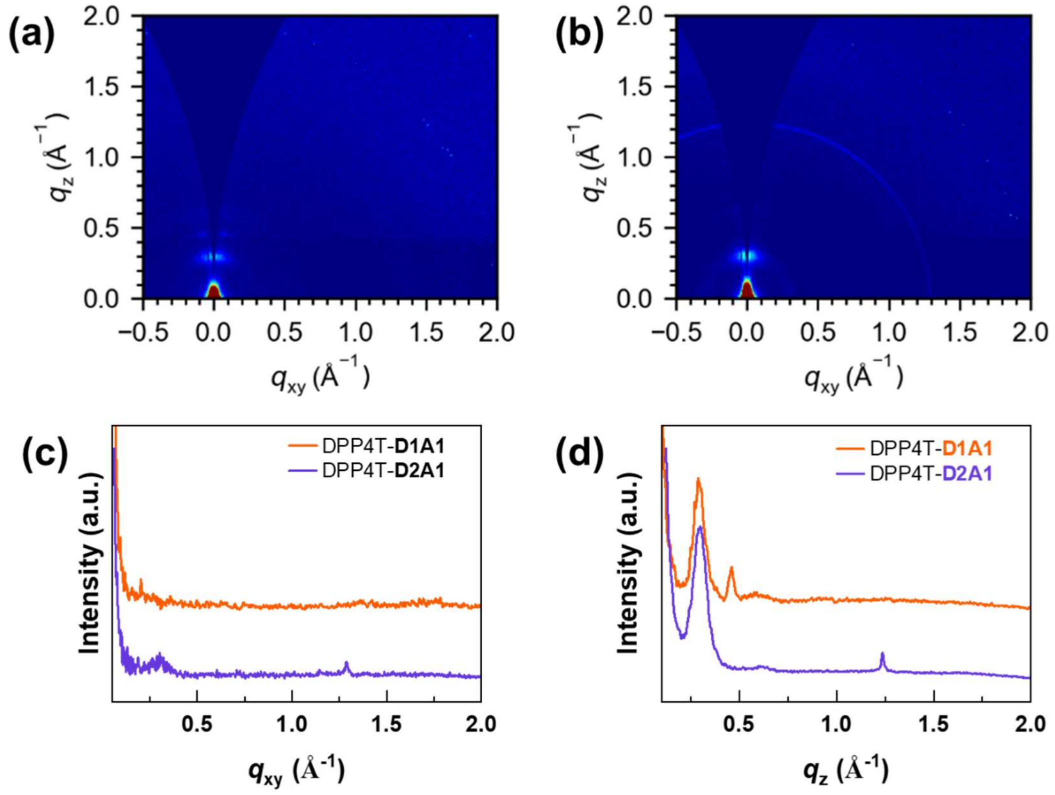

Figure S10. GIWAXS patterns of DPP4T-D1A1 (a) and DPP4T-D2A1 (b) film. Line cuts in the in-plane (c) and out-plane (d) direction.

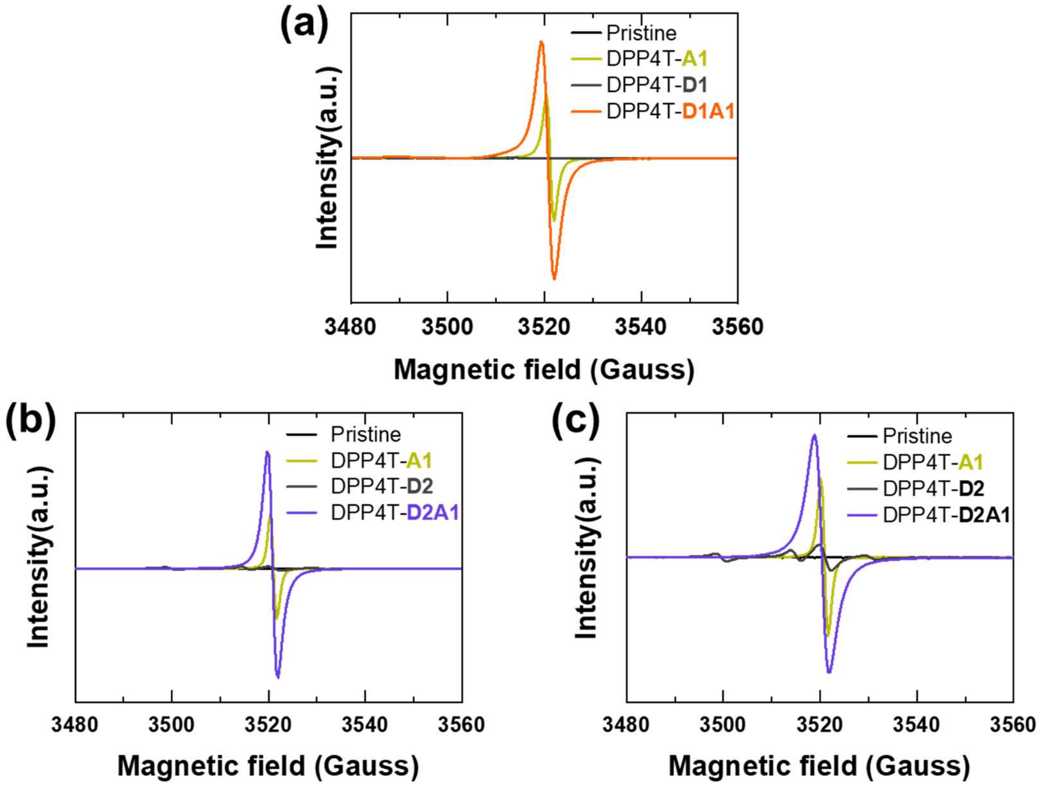

Figure S11. ESR spectra of pristine DPP4T film and doped DPP4T-D1, DPP4T-A1, DPP4T-D1A1 film at each maximum electrical conductivity (a); ESR spectra of pristine DPP4T film and doped DPP4T-A1, DPP4T-D2, DPP4T-D2A1 film at dopant molar ratio of 0.1 (DPP4T-D2A1 has a positive Seebeck coefficient) (b); ESR spectra of pristine DPP4T film and heavily doped DPP4T-A1, DPP4T-D2, DPP4T-D2A1 film at dopant molar ratio of 1.1 (DPP4T-D2A1 has a negative Seebeck coefficient) (c).

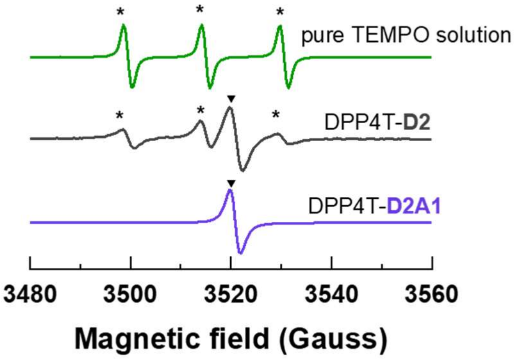

Figure S12. ESR spectra peak comparison of doped DPP4T-D2, DPP4T-D2A1 film, and TEMPO toluene solution.

It indicates that DPP4T-D2 generated TEMPO free radicals when D2 only was used,[14] and TEMPO free radicals signal was not observed in DPP4T-D2A2 when D2 was used with A1, that may be attributed to unpaired-electron free $TEMPO^{-}$ anion species generated through a double doping process of D2.[15,16]

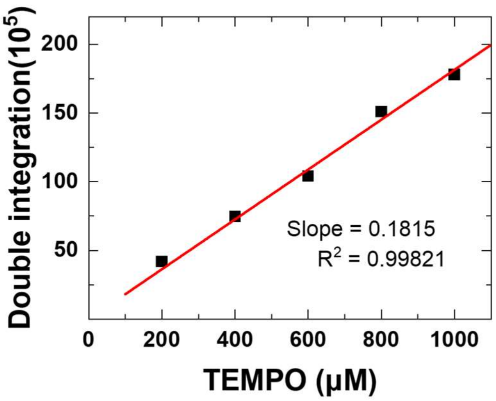

Figure S13. Linear fitting result of external standard TEMPO concentration and double integration value in quantitative ESR experiment.

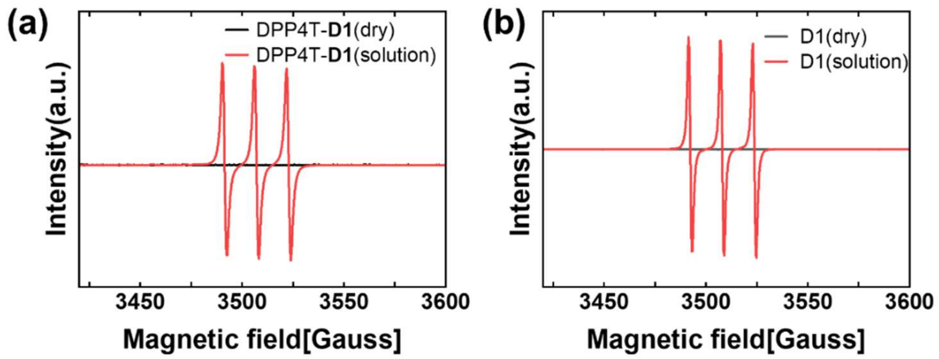

Figure S14. The volatility of D1 verified by ESR. (a) EPR signal of DPP4T-D1 in solution and dry film; (b) pure TEMPO in solution and dry on a substrate.

It is unusual that DPP4T-D1 film did not show a signal in the electron paramagnetic resonance (EPR)spectroscopy data since TEMPO(D1) originally had an EPR signal. Additional experiments were performed to test the EPR of DPP4T-D1 and D1 only in both solution and dry status. Both DPP4T-D1 and D1 only in solutions had strong signals which were assigned to the TEMPO radical, while DPP4T-D1 and D1 only dry films showed no signal. The results demonstrated that DPP4T would not quench TEMPO radical. It was hypothesized that the TEMPO had been evaporated in the dry films since the boiling point of TEMPO (193˚C) was close to that of the used solvent (179˚C) (boiling point data obtained from www.chemspider.com of The Royal Society of Chemistry).

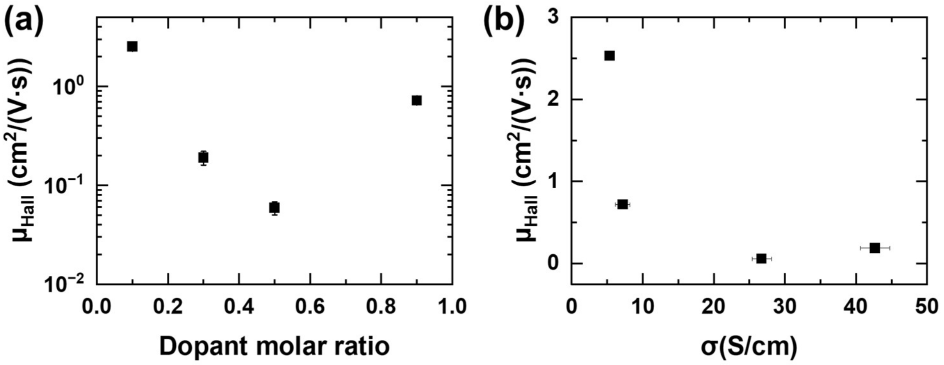

Figure S15. Hall effect measurement. (a) Hall mobility μ_{Hall} of DPP4T-D2A1 as a function of the different dopant molar ratio; (b) Hall mobility versus the electrical conductivity. Error bars were standard deviations from at least 3 sample.

The carrier mobility of DPP4T-D2A1 film was in the range of 0.06-2.5 $cm^2/(V \cdot s)$, which decreased with the increase of the dopant molar ratio since the dopants were non-conductive materials. Then, it increased at heavily doping level with the dopant molar ratio of 0.9. The rise of the carrier mobility should be due to formation of the crystalline regions as suggested by Graham et. al.[17] The electrical conductivity of DPP4T-D2A1 increased with the decrease of the carrier mobility, which was also commonly reported in previous works since the electrical conductivity of polymeric materials was affected by both the carrier mobility and the carrier concentration that was related to the efficient molecular doping level.[18-22]

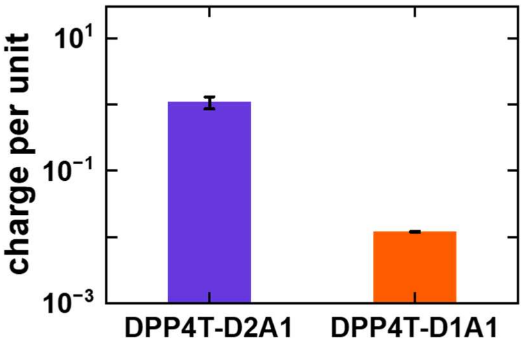

Figure S16. Charge per repeated unit of doped DPP4T count by Hall carrier concentration. Error bars were standard deviations from at least 3 sample.

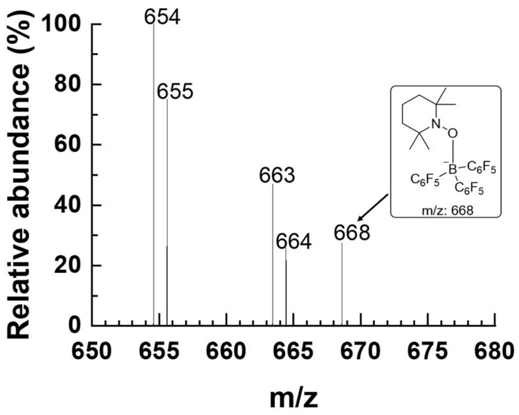

Figure S17. Negative scan results of DPP4T-D2A1 doping reaction product analysis by ESI-MS.

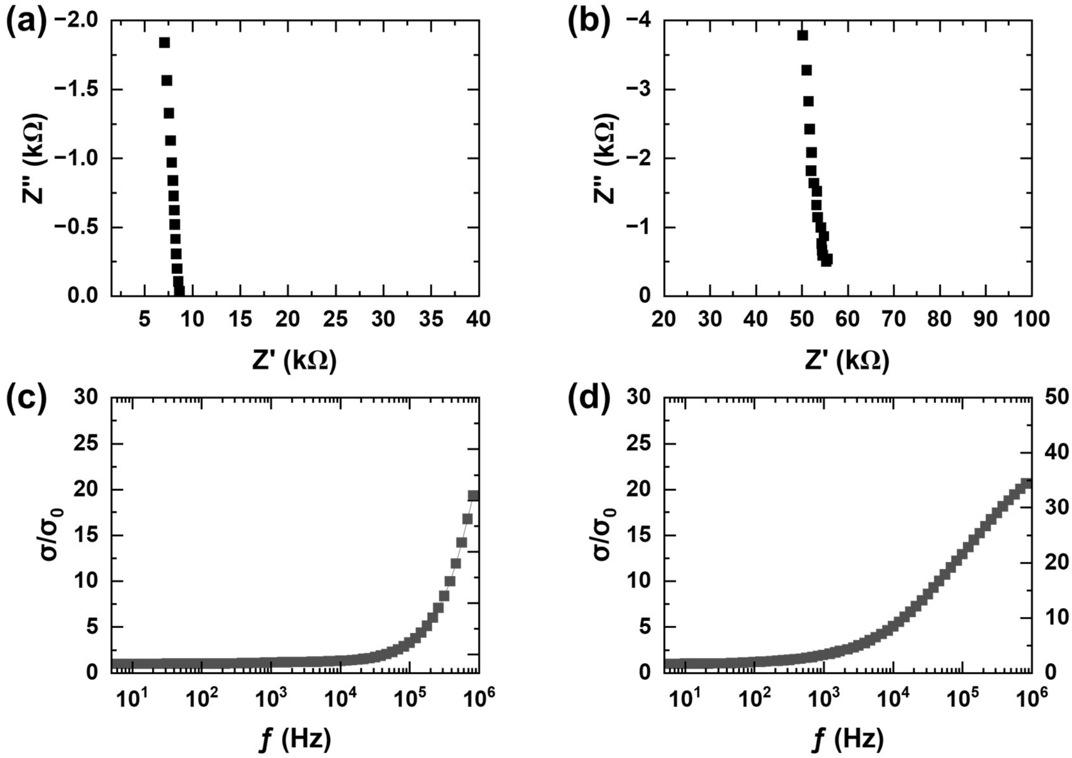

Figure S18. Electrochemical impedance spectroscopy measurement. The intercept of the Nyquist plots horizontal axis at 0.3(a) and 1.3(b) dopant molar ratio; The change of conductivity at different frequencies of DPP4T-D2A1 at 0.3(c) and 1.3(d) dopant molar ratio.

The Nyquist plots of DPP4T-D2A1 films at the dopant molar ratio of 0.3 (p-type) and 1.3 (n-type) showed data regrouped around a point (like for an ideal resistance), indicating a dominant electronic conduction of these films.[23,24] The normalized electrical conductivities for the films were constant up to 10^4 and 10^3 Hz for the dopant molar ratio of 0.3 (p-type) and 1.3 (n-type), respectively. Indicating that electronic transport was dominated over ionic effects since the increase of the electrical conductivity often occurred at a very low frequency (about 0.3 Hz).[25-27] Therefore, the ionic effects on the Seebeck coefficient and the electrical conductivity in the films may be ignored

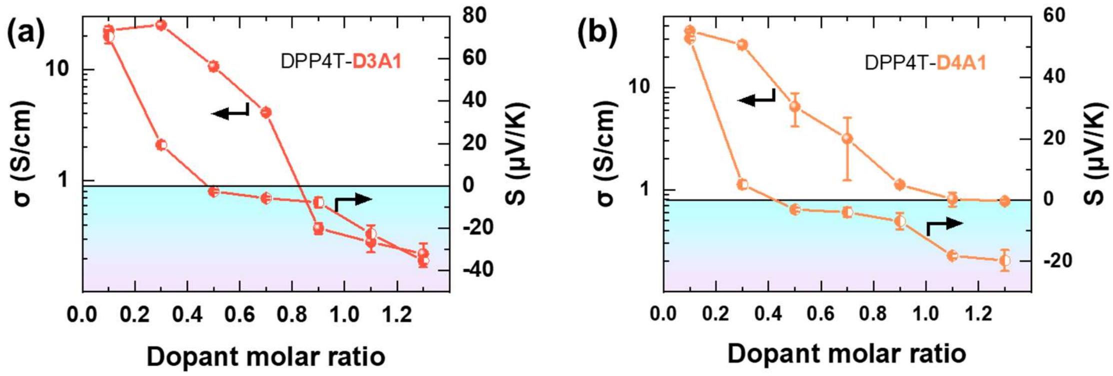

Figure S19. Coupled raction doping of DPP4T-D3A1 and DPP4T-D4A1. Electrical conductivity and Seebeck coefficient of doped DPP4T as a function of dopant molar ratio when dopant was changed to other molecule structure D3 (a) and D**4** (b) (with additive A1). both DPP4T-D3A1 and DPP4T-D4A1 films exhibit n-type Seebeck coefficient at high dopant molar ratio. Error bars were standard deviations from at least 3 sample.

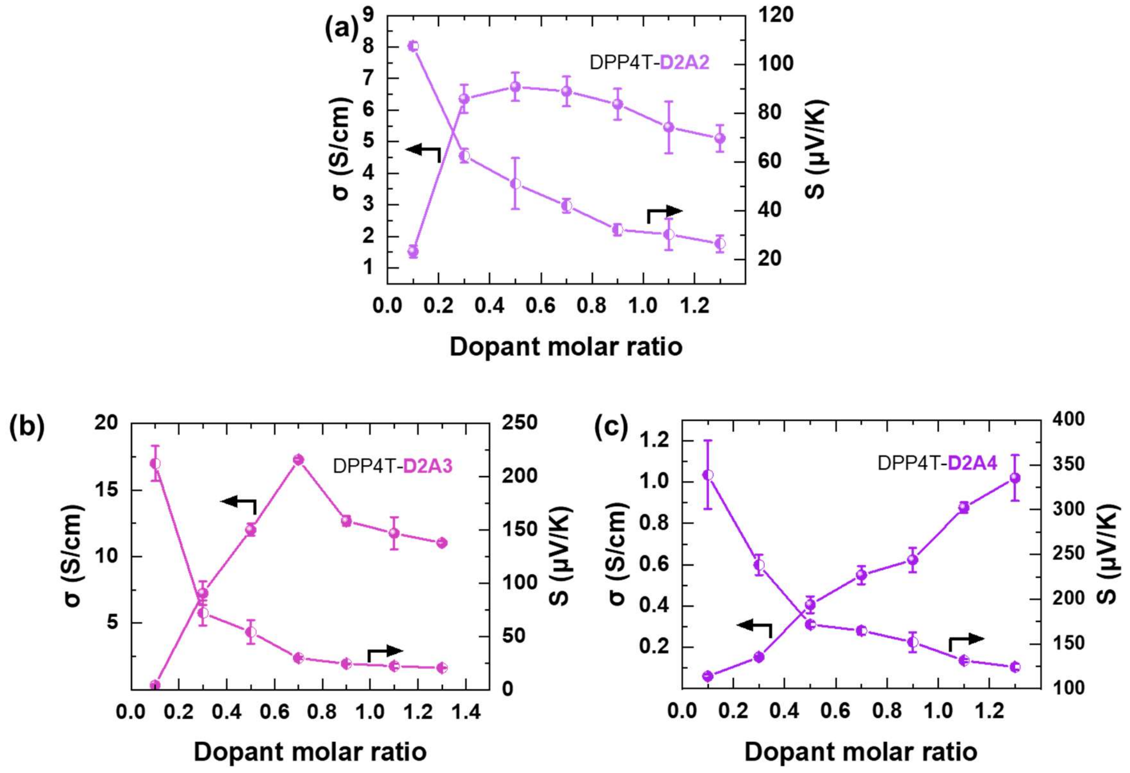

Figure S20. Coupled raction doping of DPP4T-D2A2, DPP4T-D2A3 and DPP4T-D2A4. Electrical conductivity and Seebeck coefficient of doped DPP4T as a function of dopant molar ratio when the additive was changed to other molecule structures A2 (a), A3 (b), and A4 (c) (with dopant D2). Error bars were standard deviations from at least 3 sample.

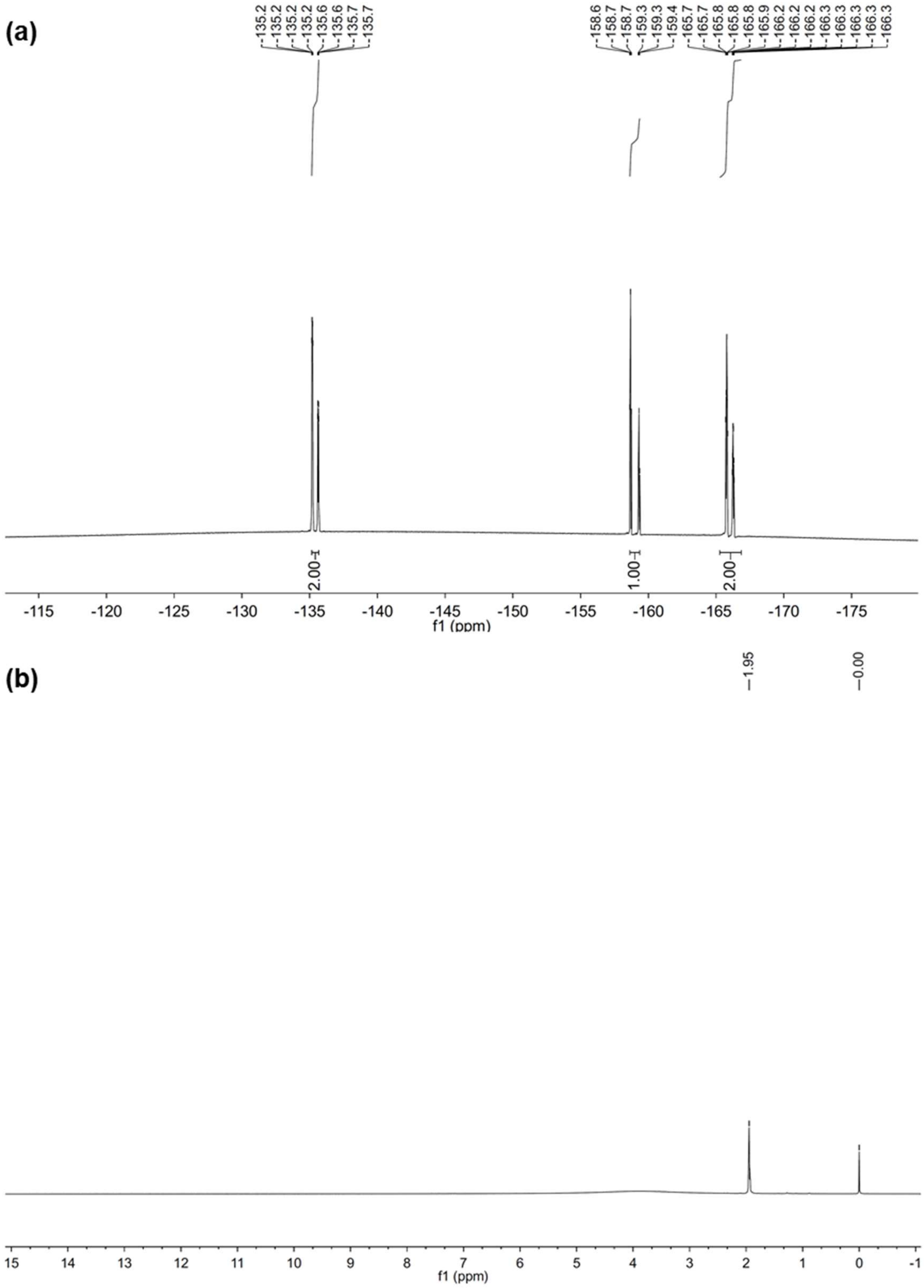

Figure S21. NMR of commercial purchased A1 to exclude the effects of impurities such as H_2O. ^{19}F-NMR (a) and ^{1}H-NMR (b) of commercial tris(pentafluorophenyl) boron (CD_3CN, 400 MHz). No other obvious peak was observed except tris(pentafluorophenyl) boron, tetramethylsilane (internal standard), and solvent peak.

Table S1. Comparison of IP/EA calculations values and the experimental values obtained with electrochemical method

BCF	– 2208.2736928 0000
BCF+e-	– 2208.3952699 4000
TEMPO	– 483.71839771 000
TEM PO+	– 483.51632922 000

A 500 meV difference between the theoretical and experimental values were commonly reported in previous literature.[28-30] In addition, the difference between the theoretical and experimental values would not change the main conclusion in this manuscript.

Table S2. Hall effect measurement result data.

	Hall Carrier concentration (cm^{-1})	Hall Mobility ($cm^2\ V^{-1}\ s^{-1}$))
DPP4T-D1A1	$5.0 \pm 0.1 \times 10^{18}$	27.05 ± 0.33
DPP4T-D2A1	$4.5 \pm 0.9 \times 10^{20}$	0.19 ± 0.03

Table S3. Max electrical conductivity data of our doping strategy compare with $FeCl_3$-doped same structure polymer

<table>
<tr><th rowspan="2">Polymer</th><th colspan="2">Electrical conductivity (S/cm)</th></tr>
<tr><th>$FeCl_3$ doping</th><th>Our doping strategy (polymer-D2A1)</th></tr>
<tr><td rowspan="2">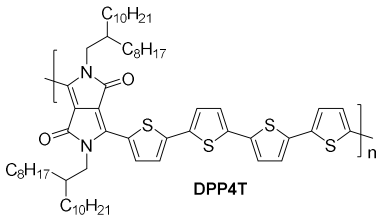 DPP4T</td><td>35 [27]</td><td rowspan="2">43</td></tr>
<tr><td>17 [32]</td></tr>
<tr><td>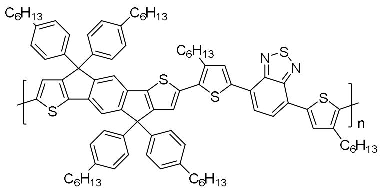 PIDT-HTBT</td><td>20 [31]</td><td>23</td></tr>
</table>

Supplementary References:

1. Zhao, W., Ding, J., Zou, Y., Di, C.-a. & Zhu, D. Chemical doping of organic semiconductors for thermoelectric applications. *Chem. Soc. Rev.* **49**, 7210-7228 (2020).
2. Wang, Y. *et al.* Tuning the Ambipolar Character of Copolymers with Substituents: A Density Functional Theory Study. *J. Phys. Chem Lett.* **11**, 3928-3933 (2020).
3. Yang, X. *et al.* Multiscale supramolecular polymer network with microphase-separated structure enabled by host–guest self-sorting recognitions. *Chem. Eng. J.* **450**, 138135 (2022).
4. Liang, Z. *et al.* n-type charge transport in heavily p-doped polymers. *Nat. Mater.* **20**, 518-524 (2021).
5. Ashraf, R. S. *et al.* Chalcogenophene Comonomer Comparison in Small Band Gap Diketopyrrolopyrrole-Based Conjugated Polymers for High-Performing Field-Effect Transistors and Organic Solar Cells. *J. Am. Chem. Soc.* **137**, 1314-1321 (2015).
6. Zhang, X. *et al.* Molecular Packing of High-Mobility Diketo Pyrrolo-Pyrrole Polymer Semiconductors with Branched Alkyl Side Chains. *J. Am. Chem. Soc.* **133**, 15073-15084 (2011).
7. Sun, B., Hong, W., Aziz, H., Abukhdeir, N. M. & Li, Y. Dramatically enhanced molecular ordering and charge transport of a DPP-based polymer assisted by oligomers through antiplasticization. *J. Mater. Chem. C* **1**, 4423-4426 (2013).
8. Holtrop, F. *et al.* Single-Electron Transfer in Frustrated Lewis Pair Chemistry. *Angew. Chem. Inter. Ed.* **59**, 22210-22216 (2020).
9. Blinco, J. P. *et al.* Experimental and Theoretical Studies of the Redox Potentials of Cyclic Nitroxides. *J. Org. Chem.* **73**, 6763-6771 (2008).
10. Jacobs, I. E. *et al.* High-Efficiency Ion-Exchange Doping of Conducting Polymers. *Adv. Mater.* **34**, 2102988 (2022).
11. Wang, J. *et al.* P-Type Chemical Doping-Induced High Bipolar Electrical Conductivities in a Thermoelectric Donor–Acceptor Copolymer. *CCS Chem.* **3**, 2482-2493 (2021).
12. Wang, J. *et al.* Lateral Fully Organic P–N Diodes Created in a Single Donor–Acceptor Copolymer. *Adv. Mater.* **34**, 2106624 (2022).
13. Kroon, R. *et al.* Polar Side Chains Enhance Processability, Electrical Conductivity, and Thermal Stability of a Molecularly p-Doped Polythiophene. *Adv. Mater.* **29**, 1700930 (2017).
14. Holtrop, F. *et al.* Single‐Electron Transfer in Frustrated Lewis Pair Chemistry. *Angew. Chem. Inter. Ed.* **59**, 22210-22216 (2020).
15. Kiefer, D. *et al.* Double doping of conjugated polymers with monomer molecular dopants. *Nat. Mater.* **18**, 149-155 (2019).
16. Leifert, D. & Studer, A. Organic Synthesis Using Nitroxides. *Chem. Rev.* **123**, 10302-10380 (2023).
17. Liang, Z. *et al.* n-type charge transport in heavily p-doped polymers. *Nat. Mater.* **4**, 518-524 (2021).
18. Chang, J.-F. *et al.* Hall-Effect Measurements Probing the Degree of Charge-Carrier Delocalization in Solution-Processed Crystalline Molecular Semiconductors. *Phys. Rev. Lett.* **107**, 066601 (2011).
19. Podzorov, V. *et al.* Intrinsic Charge Transport on the Surface of Organic Semiconductors. *Phys. Rev. Lett.* **93**, 086602 (2004).
20. Uemura, T. *et al.* Temperature dependence of the Hall effect in pentacene field-effect transistors: Possibility of charge decoherence induced by molecular fluctuations. *Phys. Rev. B* **85**, 035313 (2012).
21. Yi, H. T., Gartstein, Y. N. & Podzorov, V. Charge carrier coherence and Hall effect in organic

semiconductors. *Sci. Rep.* **6**, 23650 (2016).

22. Choi, H. H. *et al.* Hall Effect in Polycrystalline Organic Semiconductors: The Effect of Grain Boundaries. *Adv. Funct. Mater.* **30**, 1903617 (2020).
23. Wang, H., Ail, U., Gabrielsson, R., Berggren, M. & Crispin, X. Ionic Seebeck Effect in Conducting Polymers. *Adv. Energy Mater.* **5**, 1500044 (2015).
24. Wang, C. & Hong, J. Ionic/Electronic Conducting Characteristics of LiFePO4 Cathode Materials: The Determining Factors for High Rate Performance. *Electrochem. Solid-State Lett.* **10**, A65 (2007).
25. Salazar, P. F., Stephens, S. T., Kazim, A. H., Pringle, J. M. & Cola, B. A. Enhanced thermo-electrochemical power using carbon nanotube additives in ionic liquid redox electrolytes. *J. Mater. Chem. A* **2**, 20676-20682 (2014).
26. Wang, H., Hsu, J.-H., Yang, G. & Yu, C. Novel Organic Schottky Barrier Diode Created in a Single Planar Polymer Film. *Adv. Mater.* **28**, 9545-9549 (2016).
27. Wang, J. *et al.* Lateral Fully Organic P–N Diodes Created in a Single Donor–Acceptor Copolymer. *Adv. Mater.* **34**, 2106624 (2022).
28. Gao, Y. *et al.* An n-Type Conjugated Polymer with Low Crystallinity for High-Performance Organic Thermoelectrics. *Angew. Chem. Int. Ed.* **63**, e202402642 (2024).
29. Shi, Y. *et al.* A High-Performance n-Type Thermoelectric Polymer from C−H/C−H Oxidative Direct Arylation Polycondensation. *Angew. Chem. Int. Ed.* **62**, e202219262 (2023).
30. Tu, L. *et al.* Cyano-Functionalized Pyrazine: A Structurally Simple and Easily Accessible Electron-Deficient Building Block for n-Type Organic Thermoelectric Polymers. *Angew. Chem. Int. Ed.* **63**, e202319658 (2024).
31. Zhong, F. *et al.* Molecular engineering accelerated polarity switching enabling high-performance n-type organic thermoelectrics. *J. Mater. Chem. A* **10**, 18030-18037 (2022).
32. Z. Liang et al., n-type charge transport in heavily p-doped polymers. *Nat. Mater.* **20**, 518-524 (2021).
