# Supplementary material for: Efficient molecular doping of polymeric semiconductors improved by coupled reaction
Source: Nat Commun. 2024 Jul 12;15:5854. doi: 10.1038/s41467-024-50293-1 (PMC11245478; doi:10.1038/s41467-024-50293-1)
Supplement: Supplementary file 3 — Description of Additional Supplementary Files [file 41467_2024_50293_MOESM3_ESM.pdf]

Description of Additional Supplementary Files

Supplementary Data 1 - Computational atomic coordinates
